# Therapeutic Renin Inhibition in Diabetic Nephropathy—A Review of the Physiological Evidence

**DOI:** 10.3389/fphys.2020.00190

**Published:** 2020-03-12

**Authors:** Bianca Domingues Massolini, Stephanie San Gregorio Contieri, Giulia Severini Lazarini, Paula Antoun Bellacosa, Mirela Dobre, Georg Petroianu, Andrei Brateanu, Luciana Aparecida Campos, Ovidiu Constantin Baltatu

**Affiliations:** ^1^Center of Innovation, Technology and Education-CITÉ, São José dos Campos Technology Park, São José dos Campos, São Paulo, Brazil; ^2^Institute of Biomedical Engineering, Anhembi Morumbi University, Laureate International Universities, São José dos Campos, São Paulo, Brazil; ^3^Division of Nephrology and Hypertension, University Hospitals, Cleveland, OH, United States; ^4^College of Medicine and Health Sciences, Khalifa University, Abu Dhabi, United Arab Emirates; ^5^Medicine Institute, Cleveland Clinic, Cleveland, OH, United States; ^6^College of Health Sciences, Abu Dhabi University, Abu Dhabi, United Arab Emirates

**Keywords:** diabetes mellitus, diabetic nephropathy, renin inhibitor, plasma renin activity, renin- angiotensin system

## Abstract

The purpose of this systematic review was to investigate the scientific evidence to support the use of direct renin inhibitors (DRIs) in diabetic nephropathy (DN). MEDLINE was searched for articles reported until 2018. A standardized dataset was extracted from articles describing the effects of DRIs on plasma renin activity (PRA) in DN. A total of three clinical articles studying PRA as an outcome measure for DRIs use in DN were identified. These clinical studies were randomized controlled trials (RCTs): one double-blind crossover, one *post hoc* of a double-blind and placebo-controlled study, and one open-label and parallel-controlled study. Two studies reported a significant decrease of albuminuria associated with PRA reduction. One study had a DRI as monotherapy compared with placebo, and two studies had DRI as add-in to an angiotensin II (Ang II) receptor blocker (ARB). Of 10,393 patients with DN enrolled in five studies with DRI, 370 (3.6%) patients had PRA measured. Only one preclinical study was identified that determined PRA when investigating the effects of aliskiren in DN. Moreover, most of observational preclinical and clinical studies identified report on a low PRA or hyporeninemic hypoaldosteronism in DM. Renin inhibition has been suggested for DN, but proof-of-concept studies for this are scant. A small number of clinical and preclinical studies assessed the PRA effects of DRIs in DN. For a more successful translational research for DRIs, specific patient population responsive to the treatment should be identified, and PRA may remain a biomarker of choice for patient stratification.

## Introduction

Diabetic nephropathy (DN) is the primary cause of chronic kidney disease. Despite therapeutic advances, DN remains the principal cause of mortality in diabetic patients (Dugbartey, 2017).

Renin–angiotensin system (RAS) has been classically involved in the progression of diabetic cardiovascular disease. A chronically activated endocrine or paracrine RAS is considered as a principal contributor to the pathophysiology of end-organ damage in diabetes mellitus (DM), including the DN (Urushihara and Kagami, 2017). As a result, therapeutic drugs for DN are targeting mostly the renin–angiotensin–aldosterone system (Yacoub and Campbell, 2015).

Although debate remains, the therapeutic drugs for DN currently consist mainly of angiotensin II (Ang II) receptor blockers (ARBs) and angiotensin-converting enzyme inhibitors (ACEIs) used for their antihypertensive and antiproteinuric measures (Bhattacharjee et al., 2016). Direct renin inhibitors (DRIs) acting on rate-limiting enzyme of the RAS offered probability of a greater inhibition of the system so as to have better therapeutic outcomes in patients with DN (Parving et al., 2008). The rationale for developing renin inhibitors was as follows: renin is the first and rate-limiting step in RAS cascade (low renin concentration in the pM range), renin has high specificity for angiotensinogen (little side effects anticipated), ACEIs and ARBs result in incomplete RAS suppression [reactive rise in plasma renin activity (PRA), “escape” mechanism, and other products of RAS (e.g., Ang1–7, AIII, and AIV)] (Wood and Close, 1996; Nussberger et al., 2002; Stanton, 2003). However, larger trials of the DRI aliskiren in combination with an ACE inhibitor or ARB in patients with DN did not reduce cardiovascular or renal outcomes (Parving et al., 2012).

Plasma renin activity played a central role as a pharmacological biomarker for drug development, safety, and dosing in the research and development (R&D) of DRIs such as remikiren, enalkiren, zankiren, and aliskiren. Generally, DRIs induced rapid reductions of 65–95% PRA (Lambers Heerspink et al., 2009). PRA has been used in the estimation of the extracellular volume, because this correlates inversely with PRA (Juncos, 2013). Hence, Brunner et al. (1972) categorized hypertensive patients by their volume status using PRA levels. Augmented PRA levels represent a risk factor of cardiovascular disease (Alderman et al., 1991; Parving et al., 2009). Whereas several pathologies are associated with an augmented PRA, DM and associated DN apparently are not.

The therapeutic effects of RAS inhibitors may be important depending on the pathological activation of endocrine and/or tissue RAS (Gasparo et al., 2013; de Alencar Franco Costa et al., 2015). For instance, disease conditions with low baseline PRA levels reduced the treatment efficacy of DRIs (Stanton et al., 2009). Few reviews on DRI for DN as therapeutic target discussed PRA as an outcome measure (Abassi et al., 2009; Rafiq et al., 2011; Jagadeesh et al., 2012). These reviews document that PRA is reduced in DM with or without DN.

Early studies described hyporeninemia or low-renin state as a characteristic state of circulatory RAS in DM patients with or without DN (Sousa et al., 2016). Our and other studies (de Alencar Franco Costa et al., 2015; Sousa et al., 2016) evidencing a diabetes-induced low-renin status may indicate that a DRI is not always effective in treating DN. Therefore, the purpose of this study was to examine and synthesize the existing literature on DRI effects on PRA in DN. Literature search included studies that investigated DRIs such as remikiren, enalkiren, zankiren, or aliskiren in DN.

## Methods

### Literature Search Strategy

We conducted a systematic review of investigative studies in accordance with the Preferred Reporting Items for Systematic Reviews and Meta-Analyses (PRISMA) consensus guidelines (Moher et al., 2016). A literature search of the MEDLINE database via PubMed was performed using a structured approach to identify relevant studies. A manual search was also conducted through searching the reference lists of relevant articles to expand the included studies. Eligibility assessment of identified articles was performed independently by two reviewers for preclinical studies and two reviewers for clinical studies, and inconsistencies were settled by one of the senior reviewers.

### Inclusion Criteria

To identify relevant articles on original research, we associated terms referring to the use of PRA and/or renin inhibitors in DN. All experimental studies on humans and animals were eligible. Document types included were those produced as original and review papers written in English and published until 2018. The following Medical Subject Headings (MeSH) were used in the search: DN OR “diabetic kidney disease” AND “renin inhibitor” OR “aliskiren” OR “remikiren” OR “enalkiren” OR “zankiren.” We used filters to select the type of study, and we gathered data from clinical trials and from case–control and cohort studies and reviews, designed to assess the effects of DRIs on PRA in DN.

### Exclusion Criteria

Articles were excluded if they were clinical case reports, clinical case series, letters, editorials, opinions, points of views, or anecdotes. Also, articles that were written in languages other than English were discarded.

### Data Extraction and Quality Assessment

Four investigators evaluated independently titles and abstracts and selected the articles for further full-text evaluation. Disagreements were resolved by consensus or by consultation with one of the senior investigators. When data were not found in the published article, authors were contacted to provide the missing information. The following data were collected: title, author and study group, publication year, DN, DRI, and PRA.

## Results

### Search Results and Study Selection

**FIGURE 1:**
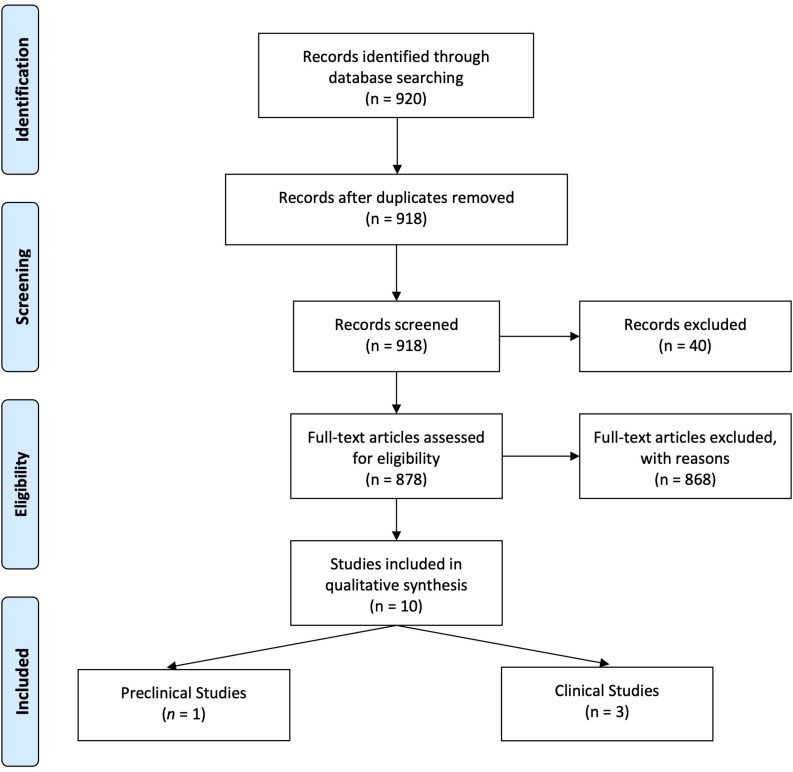
PRISMA Flow Diagram.

Figure 1 details the search and selection process of articles that determined (or discussed in case of reviews) PRA when investigating DRI effects in DN. Of 920 potentially relevant papers initially identified through the PubMed search, after de-duplication, we reviewed 918 titles and abstracts; from these, we included 878 in a full-text review. A further 873 articles were excluded after full review, and five were included in the present study: one preclinical study and four clinical studies.

### Clinical Studies With Direct Renin Inhibitor in Diabetic Nephropathy That Determined Plasma Renin Activity

Three clinical studies have been identified to have reported effects of DRI (aliskiren) on PRA in DN (Table 1). Two studies reported a significant decrease of albuminuria associated with PRA reduction (Persson et al., 2009; Abe et al., 2012). The *post hoc* analysis of ALTITUDE study (Parving et al., 2012) in a subset of 133 patients reported a non-significant reduction of urinary albumin creatinine of 22 and 9% in the aliskiren and placebo groups, respectively (Persson et al., 2012a).

**TABLE 1:** Clinical Studies with DRI in diabetic nephropathy that determined plasma renin activity.

**Study**	**Study type**	**DRI monotherapy/add-in therapy**	**Study participants**	**Renal outcomes**	**DRI effect on PRA**
Persson et al., 2009	Double-blind, randomized, crossover trial	Aliskiren, irbesartan, and aliskiren/irbesartan, 2-month treatment	26 patients with T2DM, HTN, and albuminuria (>100 mg/day)	Significant reduction in urinary albumin, glomerular filtration rate, and 24-h blood pressure from placebo	72%↓ as monotherapy compared with placebo
Persson et al., 2012a	AVOID *post hoc* analysis	Add-in: aliskiren or placebo in addition to losartan, 6-month treatment	Patients with HTN and T2DM with nephropathy: a prespecified subset of 133 (22%) patients from a total of 599 patients	Not significant reduction in urinary albumin–creatinine ratio	71%↓ compared with placebo (90%↓ compared with baseline; placebo: 19%↓)
Abe et al., 2012	Open-label, randomized, parallel-controlled study	Add-in: aliskiren or placebo in addition to telmisartan and amlodipine, 6-month treatment	64 patients with T2DM, DN, and HTN	Significant reduction in urinary albumin–creatinine ratio	70–77%↓ compared with baseline; 89%↓ compared with calcium channel blocker (CCB) group

*T2DM, type 2 diabetes mellitus; DN, diabetic nephropathy; HTN, hypertension; PRA, plasma renin activity; DRI, direct renin inhibitor.*

One double-blind, randomized study that investigated the effect of aliskiren as monotherapy in patients with DM and hypertension (HTN) reported a decrease of 72% in PRA (Persson et al., 2009). Two other studies that investigated aliskiren or placebo in addition to an ARB [one a *post hoc* analysis (Persson et al., 2012a) and the other an open-label, randomized study (Abe et al., 2012)] reported a PRA decrease of 71–77%. The data from the studies were heterogeneous and not sufficient to carry out a quantitative analysis. There were not enough data in two studies (Abe et al., 2012; Persson et al., 2012a), the reported PRA data had a skewed distribution in one study (Persson et al., 2009), and there was no blindness in one study (Abe et al., 2012). In addition, in one study, PRA was determined only in a subset of patients from the total investigated in the aliskiren group: 22% (133 of 599) patients in the (Persson et al., 2012a) study.

Of 10,393 patients with DN enrolled in five studies [599 in Parving et al. (2008); 26 in Persson et al. (2009); 8,561 in the ALTITUDE study (Parving et al., 2012); 64 in Abe et al. (2012); 1,143 in the VIvID study (Bakris et al., 2013)], 370 (3.6%) patients had PRA measured (Persson et al., 2009, 2012a; Abe et al., 2012).

### Preclinical Studies With Direct Renin Inhibitor in Diabetic Nephropathy That Determined Plasma Renin Activity

One preclinical proof-of-concept study testing the effects of aliskiren in DN determined PRA (Table 2). This study used as model for DM the streptozotocin (STZ)-induced DM in C57BL/6J mice fed on a high-fat diet, determined PRA, and found higher levels in DN when compared with the control non-DN (Kidokoro et al., 2016). In Table 2 are included articles that reported renal renin outcome measures, including plasma renin concentration and renin mRNA expression.

**TABLE 2:** Preclinical studies with DRI in diabetic nephropathy that determined plasma renin or renal RAS.

**Study**	**Experimental model**	**DRI**	**Outcome**	**Renal/plasma renin in DN control vs. healthy control**	**Effects of DRI on renal/plasma renin**
Dong et al., 2010a	db/db mice, with obesity and T2DM	Aliskiren, 6 weeks’ treatment	Protects against cardiovascular complications and pancreatic injury	Renal renin mRNA not different than that of control db/m mice	Increased renal renin mRNA expression
Dong et al., 2010b	db/db mice with obesity and T2DM	Aliskiren, 6 weeks’ treatment	Protects against DN	Renal renin mRNA higher than that in control db/m mice	Increased renal renin mRNA expression
Kang et al., 2011	db/db mice with obesity and T2DM	Aliskiren, 3 months’ treatment	Decreased albuminuria, glomerulosclerosis, interstitial fibrosis, improved insulin resistance	Lower plasma renin concentration (PRC) in db/db mice than in db/m mice (control non-DM)	Increased PRC
Wang et al., 2014	STZ-DBA/2J mice fed on a high-fat diet	Aliskiren, 6 weeks’ treatment	Protects against DN	Renal renin mRNA in DN higher than that in control non-DN	Increased renal renin mRNA expression
Zhou et al., 2015	db/db mice, with obesity and T2DM + uninephrectomy	Aliskiren, 4 weeks’ treatment	Protects against DN	PRC normal, renal renin mRNA higher than that in control non-DN	Increased PRC and renal renin mRNA expression
Kidokoro et al., 2016	STZ-C57BL/6J mice fed on a high-fat diet	Aliskiren, 4 weeks’ treatment	Protects against DN	PRA and *in vivo* imaging of renal renin activity higher than that in control non-DN	Decreased PRA and *in vivo* imaging of renal renin activity

*T2DM, type 2 diabetes mellitus; DRI, direct renin inhibitor; RAS, renin–angiotensin system; DN, diabetic nephropathy.*

## Discussion

The present study shows that a low number of preclinical and clinical studies with DRIs as monotherapy or add-in therapy in DN assessed PRA. Only two randomized controlled studies reported renoprotective effects in DN associated with a significant reduction in PRA.

Of eight publications identified to report DRI effects on urinary albumin in DN (Parving et al., 2008, 2012; Persson et al., 2009, 2010, 2011, 2012a,b; Abe et al., 2012), only three clinical studies presented data on PRA. All three involved patients with both DM and HTN. As aliskiren does not lower blood pressure in hypertensive patients with low PRA (Sealey and Laragh, 2009), Jagadeesh et al. (2012) suggested that “it may be useful to dichotomize RAAS-related pathologic syndromes into ones associated with high renin (some HTN, any HTN after diuretic treatment), where aliskiren appears to be quite effective, and low-renin” (like diabetes), where aliskiren is of uncertain value. Indeed, in the AVOID study (Parving et al., 2008), a prespecified subset of 133 (22%) patients from a total of 599 patients was identified with a significant decrease in PRA by aliskiren (Persson et al., 2012a). The study of Uresin et al. (2007) that reported a significant reduction in 24-h ambulatory blood pressure presented PRA data on 32% of the total patients recruited in the DRI study group. As such, important information that could lead to patient stratification could be learned from disclosing the PRA data from the ALTITUDE study (Parving et al., 2012).

Dual therapy of the DRI aliskiren with ACEI or ARBs was commonly investigated in patients with HTN, heart failure, and diabetes with or without proteinuria. It is conceptualized that the antihypertensive efficacy of aliskiren is increased when adding ACEIs, ARBs, or diuretics, which produce a reactive increase in PRA. Indeed, aliskiren in combination with ACEIs or ARBs showed significant blood pressure and proteinuria reductions than monotherapy alone in phase II trials with hypertensive patients with or without DM (Persson et al., 2009), reviewed by Şen et al. (2013). Clinical trials that studied the combination of aliskiren and ACEI or ARBs and involved patients with DM include Pool 2007, ALOFT 2008, ALLAY 2009, AVANTE GARDE 2010, VANTAGE 2010, and ASPIRE 2011. These studies were systematically reviewed by Harel et al. (2012). They were not designed to investigate outcomes in DM patients. A meta-analysis of 13,395 patients with diabetes showed no benefit from the addition of aliskiren to standard medical therapy (Zheng et al., 2017).

Finding a preclinical experimental model for DM and DN was challenged by the high selectivity of aliskiren for human renin compared with renin from other species (IC50 values [50% inhibitory concentrations]: human 0.6, marmoset 2, rat 80, dog 7) (Wood et al., 2003). The first studies of DRI on an experimental DM model used the STZ-induced DM in high-renin hypertensive (mRen-2)27 rats (Kelly et al., 2007; Feldman et al., 2008). However, this DM model cannot consider the phenotype alterations as primarily induced by DM because these rats genetically activated renin production. Four studies reported DRI effects on DN of db/db mice (Table 2). Another study on db/db mice showed no significant differences in their PRA compared to control db/m mice (Gallo et al., 2016).

Experimental models investigated for proof-of-concept efficacy of aliskiren in DN were db/db mice for type 2 DM (T2DM) and STZ mice (DBA/2J and C57BL/6J strains) for type 1 DM (T1DM). The db/db mice are characterized by T2DM, elevated systolic blood pressure, obesity, and hyperlipidemia. They develop T2DM with high plasma levels of insulin and glucose at weeks 9–10 of age (Forbes and Cooper, 2013). The main outcomes studied for DN, including increased albumin excretion and glomerular pathology, are very similar between mouse lines with T1DM or T2DM and humans with DM (Azushima et al., 2017).

**FIGURE 2:**
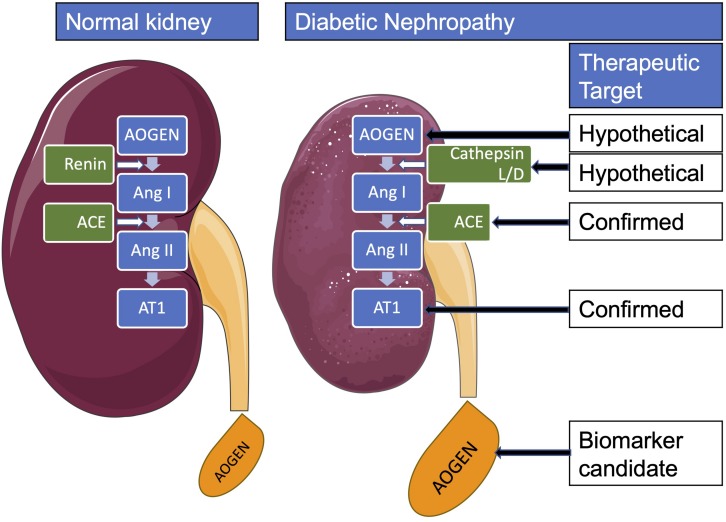
Renal renin-angiotensin system in diabetes mellitus. AOGEN, angiotensinogen; ACE, angiotensin-converting enzyme; Ang, angiotensin; AT1, angiotensin type 1 receptor.

Locally activated synthesis and activity of renin have been identified in kidney and other organs in different pathologies (Bader et al., 2001). Such organs where a local tissue RAS has been postulated include the heart, blood vessels, kidney, brain, adipose tissue, adrenal gland, pancreas, liver, reproductive system, lymphatic tissue, placenta, and eyes (Nehme et al., 2019). Diseases where chronically activated local tissue RAS has been identified include HTN, atherosclerosis, heart failure, cardiac hypertrophy and fibrosis, chronic kidney disease, and glaucoma (Ames et al., 2019; Nehme et al., 2019). An increase in local production of active angiotensins could be through the classical renin–ACE pathway or through alternative pathways (Ferrario et al., 2014). Translational proof-of-concept studies shall distinguish the enzymes involved in these RAS pathways in order to identify therapeutic targets. For instance, we have demonstrated in a proof-of-concept study that renin might be a therapeutic target in glaucoma (Wang et al., 2012). This does not seem to be the case in DN where both preclinical and clinical proof-of-concept studies indicate that DN is associated with a low-renin state. In Table 3B, we summarized the clinical studies that described hyporeninemia in DM, with the first report dated year 1973. These studies indicate that hyporeninemic hypoaldosteronism is underdiagnosed in DM (Sousa et al., 2016). Several mechanisms have been suggested as responsible for the reduction in renin release in patients with DM, including juxtaglomerular injury, autonomic dysfunction, and primary increase in renal salt retention with volume expansion (Phelps et al., 1980; Sousa et al., 2016). The first experimental studies of RAS in DM used alloxan-DM or STZ-DM rat models (Table 3A). Preclinical evidence for an activated renal RAS in DM is suggested by our and others studies on increased synthesis and urinary secretion of renal angiotensinogen (Zimpelmann et al., 2000; Saito et al., 2009; de Alencar Franco Costa et al., 2015; Lee et al., 2017) (Figure 2 shows urinary angiotensinogen as a potential biomarker). STZ-induced DM in rats caused a 69% increase of Ang II in the renal interstitial fluid, which was decreased 27% by aliskiren (6 weeks’ treatment) (Matavelli et al., 2012). As aliskiren did not normalize the DM-increased renal interstitial fluid Ang II, alternative Ang II-forming pathways might have been activated. One candidate enzyme that may take over the renin activity in kidney to activate the local Ang II production is cathepsin L. Cathepsin L was identified as a potential sex-specific biomarker for renal damage by the Actelion group (Bauer et al., 2011). Cathepsin L appears to be importantly involved in the development of albuminuria and renal damage in early experimental DN (Garsen et al., 2016) (Figure 2). Angiotensinogen may be degraded by cathepsins including cathepsin L, which may degrade angiotensinogen (Watanabe et al., 1989). As cathepsin L can be involved in the pathogenesis of DN through several mechanisms, targeting with suitable antagonists may hold promises for therapeutic interventions (Kumar and Anders, 2016). PRA is not a good indicator of local RAS activity as measure circulating production of angiotensin I, where ACE, alternative Ang II-forming enzymes, and Ang II might be increased in the local tissue. Although PRA is a pharmacological efficacy biomarker for aliskiren and has been considered as an outcome measure in DM, the effects of renin inhibitors on local tissue RAS are not easily demonstrable in clinical studies because there are no available biomarkers for local RAS activation. Studies on urinary peptidome might lead to the characterization of biomarkers for local renal RAS activation, such as cathepsin L or D (Krochmal et al., 2017).

**TABLE 3A:** Preclinical studies that determined PRA in experimental DM.

**Study**	**Experimental model**	**DM effect on PRA**
Christlieb, 1974	Alloxan-DM rat, acute DM (alloxan is nephrotoxic)	Low PRA
Christlieb et al., 1979	Alloxan-DM rat, 3 months	PRA decreased progressively
Ballermann et al., 1984	STZ-DM rat, 1 month	PRC values in untreated DM rats were lower than those of insulin-treated rats or controls
Pratt et al., 1985	Alloxan-DM rat, 7 weeks	Low PRA
Kigoshi et al., 1986	STZ-DM rat, 1.5 months	Hyporeninemic hypoaldosteronism
Kim, 1994	STZ-DM rat, 2 months	Hyporeninemic hypoaldosteronism
Di Loreto et al., 2004	Alloxan-DM rat, 1 month	Low PRA, glucose overload did not significantly affect these values
de Alencar Franco Costa et al., 2015	STZ-DM rat, 3 months	Low PRA, PRC, and renal renin mRNA

*PRA, plasma renin activity; DN, diabetic nephropathy; DM, diabetes mellitus; STZ, streptozotocin.*

**TABLE 3B:** Clinical studies on low PRA in DN.

**Study**	**Study participants**	**PRA in DM**
Chelaghma and Oyibo, 2018	One patient with DM	Hyporeninemic hypoaldosteronism
deLeiva et al., 2010	Two patients with DM	Hypoaldosteronism due to low PRA
Hollenberg et al., 2003	31 Patients with T1DM	Intrarenal RAS activated without PRA
Bojestig et al., 2000	80 Patients with T1DM	Low PRA and high plasma ANP
Elisaf et al., 1999	One patient with DM	Hyporeninemic, hypoaldosteronism, and autonomic neuropathy
Bonnet and Thivolet, 1996	One patient with DM	Hyporeninemic hypoaldosteronism
Fukuchi, 1991	118 Patients with DM	Hyporeninemic selective hypoaldosteronism may be associated with DM nephropathy or DM neuropathy
Paulsen et al., 1989	100 Teenage patients with T1DM	Decline of PRA over 5 years
Villoria et al., 1988	13 Patients with DM and chronic renal failure	Hyporeninemic hypoaldosteronism associated with type IV renal tubular acidosis
Antonipillai et al., 1981	12 Patients with DM	Low PRA and active renin (AR)
Fernandez-Cruz et al., 1979	16 Normotensive diabetics with long-term disease	Hyporeninemia
Farese et al., 1980	Four patients with DM	Diabetic hyporeninemic hypoaldosteronism
Tuck et al., 1979	Five patients with DM with mild renal insufficiency	Hyporeninemic hypoaldosteronism associated with DM and neuropathy may be due to decreased sympathetic nervous system activity
Kuhlmann et al., 1978	Three patients with DM	Hyporeninemic hypoaldosteronism
Christlieb et al., 1978	44 Patients with DM	Hyporeninemic hypoaldosteronism is frequent in diabetics with nephropathy
Christlieb et al., 1976	48 Patients with DM	(1) PRA is normal in normotensive diabetics (2) Patients with diabetes, hypertension, and nephropathy have “low renin hypertension”
Christlieb et al., 1974	Eight patients with DM	Low PRA
de Chatel et al., 1977	60 Patients with DM	Low PRA
Perez et al., 1977	12 Patients with DM	Hyporeninemia and hypoaldosteronism
Weidmann et al., 1973	Four patients with DM	Low PRA

*PRA, plasma renin activity; DN, diabetic nephropathy; DM, diabetes mellitus; RAS, renin–angiotensin system; ANP, atrial natriuretic peptide; T1DM, type 1 diabetes mellitus.*

## Conclusion

Very few studies addressed the PRA as the outcome measure of DRI treatment effect in DN. Therefore, for a more successful translational research, specific patient population where DRI treatment is effective in DN should be identified. Additional well-designed randomized controlled trials (RCTs) using PRA as a marker for patient stratification and randomization may be warranted.

## Author Contributions

OB and LC contributed to the conception and design of the study. MD, AB, GP, OB, and LC analyzed and interpreted the data. MD, AB, GP, OB, and LC drafted the manuscript. All authors provided critical revision of the article.

## Conflict of Interest

The authors declare that the research was conducted in the absence of any commercial or financial relationships that could be construed as a potential conflict of interest.
